# Plastic Operations in Carcinoma of the Breast*Read before the Southern Surgical and Gynecological Association at Charleston, S. C. November, 1894.

**Published:** 1894-12

**Authors:** J. T. Wilson

**Affiliations:** 116 N. Travis Street; Sherman, Tex.


### Southern Medical Record.

A MONTHLY JOURNAL OF MEDICINE AND SURGERY.

Vol. XXIV. ATLANTA, GA., DECEMBER, 1894. No. 12.

### Original Articles.

### PLASTIC OPERATIONS IN CARCINOMA OF THE BREAST.*

By J. T. WILSON, M. D., Sherman, Tex.

While statistics would lead us to believe that carcinoma of the breast is not so common in this country as in England, and in some other countries; nevertheless, its frequency here, and its fatality, too, is sufficient to demand our best attention and careful study.

I believe it is now agreed among a majority of scientific writers that it is primarily a local disease; that being true, there is no reason why early removal should not cure it. It is, therefore, all important to make a diagnosis at the earliest possible moment and operate before the glands become involved.

Perhaps in a majority of cases when our attention is first called to it, the disease has made considerable headway, involving the surrounding glands and probably showing the peculiar taint; in fact, some authors believe the constitution becomes involved almost simultaneously with the glands.

It has been observed that metastases occur very early in cancer of the breast, which is another reason for immediate operation.

*Read before the Southern Surgical and Gynecological Association at Charleston, S. C. November, 1894.

It has been clearly demonstrated that the patient has been rendered more comfortable and life prolonged, even in a late stage of the disease, when the tissues have been broken down and suppurating, with the neighboring glands much enlarged and the general health greatly undermined.

As soon as the diagnosis is made or even if there is a suspicion of cancer, it is time to remove the tumor.

It is very generally conceded that if left undisturbed, it invariably progresses to a fatal issue. The time usually given for it to run its course is from thirty to thirty-six months.

The younger Gross declared that the operation when it does not cure, adds ten months to the life of the patient, but that it definitely cures 11-83 per cent. of all cases, and it is safe from reproduction, if three years have elapsed since the operation.

Bilroth taught that recurrences after a certain length of time, say from eighteen months to two years probably, and three years certainly, may be regarded as a new growth, and as absolutely independent of the original growth and arises from a cancer diathesis. He thinks the scar furnishes the condition favorable to development.

Halsted differs from this view and thinks that liberation of cancer cells from their alveoli or from the lymphatic vessels may start a new cancer, and thinks this theory more plausible in accounting for the late recurrences instead of the cancer diathesis. It is well known by all who have had some experience with cancer of the breast that it does return in a majority of cases be the cause what it may. My attention was first attracted to this subject of recurrence by witnessing an operation by the elder Gross, it being the sixth upon the same patient.

I believe that all cancerous tumors of the breast should be removed no matter what stage they are in if the patient has sufficient strength and vitality to rally from the anæsthetic shock even though it is almost certain the disease will recur. It relieves the patient from pain and the disagreeable odor of the discharge if suppurating, it brings a certain amount of comfort if only for a short season, frees her mind for the time from the oppression and anxiety of the disease and it gives the patient a chance, it matters not how slender, from immunity of recurrence, and the newer operations give a much more hopeful prognosis than the old. If the wound should heal as it most frequently does, it leaves the patient more hopeful and stimulates her with the belief that she is cured and by reliev-ing her of the anxiety and distress of mind, gives her a better chance to build up her general health. There is no doubt that tranquillity of mind has a marked influence over the general health.

I am of those who believe in preparing the system for an operation generally, but in carcinoma, little time should be lost in this regard, for the sooner after its discovery the tumor or disease is removed, the more favorable is the result likely to be. Even a few days sometimes makes considerable difference. As the poison becomes diffused in the surrounding glands and tissues, the entire system is likely soon to become involved and the prognosis correspondingly grave.

The operation for removal of a cancerous breast has been modified by several eminent surgeons. The old elliptical incision once so popular is not so closely adhered to now. Prof. S. W. Gross made a circular incision, removing the gland and its covering integument, other operators are guided by existing conditions. Another point of decided importance is the removal of the axillary and other neighboring glands. I think a majority of surgeons at the present day following the suggestion of Kuster are advising the removal of these glands in every case whether they seem involved or not, some of whom assert that often their involvement cannot be known until they are cut down upon and examined, and several begin their operations by first attacking the axilla, giving as a reason that if the mamma is first removed the necessary manipulation may squeeze the fluid up the lymph channels and thus infect the surrounding glands and tissues.

Volkmann discovered cancer cells on the fascia of the great pectoral muscle under the microscope when it first appeared healthy to the naked eye, and removed it entire, even stripping the muscle of its delicate sheath.

Halsted goes even farther and removes the greater portion of this muscle, part of the pectoralis minor, the loose tissue connecting the supra and infra clavicular glands and stripping the subclavian and axillary vessels of their investing tissue.

Halsted's operation is the most complete of any surgeon's with which I am acquainted.

Haidenhain believes that even though the tumor be freely movable, it has already advanced through fat and cellular tissue to the surface of the muscle.

The thoroughness of the operation in every case is of supreme importance; the entire mammary gland, every nodule, gland and tissue whether cellular or muscular to which is attached a suspicion of involvement should be removed with the utmost care, and all skin investing the tumor should be likewise cut away. It will not do as was often done in former times to dissect up the skin and leave it for a flap, but it should be removed to the entire extent of covering the tumor in every case.

In many of these operations, in fact, in nearly all, the flap cannot be brought together without considerable stretching even when the skin has been loosened from its attachment for an inch or more around and in many cannot be stretched enough to cover the wound, which leaves a granulating surface and sometimes a large one. The sutures that are stretched and tight, in attempting to coaptate the edges of the skin, cut through to a greater or less degree, enlarging their tracks and by the great tension constricting much of the surface, interfering with the circulation and nutrition and thus impeding the healing.

The recurrence of these tumors often takes place in the cicatrix. Prof. Gross, the younger, who devoted much time to the study of this subject, thought it never recurred from the granulating surface and states that "it is a histological fact that granulation tissue will give rise to granulation tissue alone and not to epithelial tissue; the granulating surface may be great or small—that has nothing to do with the recurrence;" and yet we know that in practice recurrence does often take place in these granulating wounds sometimes before they are healed over as well as in the cicatrix. It is true that this may be due to the fact that all the tissue containing cancer cells has not been removed.

It has occurred to me that when a large amount of integument has been removed as is sometimes necessary, the attempt to bring the edges of the skin in apposition requiring very considerable stretching and leaving considerable muscular surface uncovered, the granulating is very slow, and there was more frequent recurrences than when the wound was covered by healthy skin and the tension not so great, other conditions being equal.

From my very limited experience, I am led to believe that by transferring a piece of healthy skin from another part of the body and implanting it over the exposed wound, the patients made better progress toward recovery, the wound healed more kindly, there was less pain and trouble and better results obtained.

The healthy skin thus implanted seems to have a salutary effect upon the adjacent tissues, it prevents irritation and relieves tension.

In doing this operation, the diseased parts should be most thoroughly cleaned in the usual antiseptic manner.

The part from whence the skin is taken for transplantation should also undergo the same careful treatment, but all strong antiseptic drugs used in this process should be thoroughly washed off.

The wounds and graft should not be irrigated with bichloride or other strong solution. In my humble judgment the application of such drugs to these wounds are injurious, they irritate the parts which do not heal kindly after it.

The skin flap should be transferred from its bed immediately to the wound when hemorrhage has been controlled and its under surface should not come in contact with anything but the wound which it is intended to cover. It can be attached to the edges of the surrounding skin by very few delicate silk sutures sterilized and far apart to prevent too great constriction, apposition made as nicely as possible and held in place by gentle pressure, the parts covered with iodoform gauze, over which is placed a thick layer of absorbent cotton, covered by rubber tissue and all confined by a soft flannel bandage.

Great care is necessary in removing the dressing on the third, fourth or fifth day, and it should be first soaked in a warm boracic solution until it comes away with the least possible traction.

To be successful, the care to be used in the details of this operation is very important; it requires to be done under the strictest antiseptic principles, and is rather tedious. The careful dissecting of all glands from the axillary and clavicular regions, the fat and cellular tissue and that part of the muscle adjacent to the tumor likely to be involved with as little loss of blood as possible is necessary to success.

The skin replacing that removed may be in one or more pieces and can be attached to the surface without a pedicle. In removing and attaching, this requires the greatest care and nicety of detail, every step to be planned before the operation is begun, with the precaution to have everything ready so that the wound may be covered without unnecessary delay after the tumor has been removed and hemorrhage controlled.

Krause reported to the German Medical Congress in 1893 a number of operations for various diseases where he covered large skin defects by flaps without pedicle and is much encouraged by his success. He does not include the subcutaneous tissue in the flap, but only the cutis and cuticle.

We need not be discouraged if after removing the dressing in three or four days, it does not present what seems to us a favorable appearance, as it takes seven or eight days—sometimes longer, for it to begin to take on new life and healthy action. It requires some time for a reorganization and union of the vessels by which the circulation can be completed and nutrition of the parts begin. Krause gives from three to six weeks for healing to take place.

*116 N. Travis Street.*

